# Supplementary figures and images for: Diagnostic performance of four hepatitis-B surface antigen conformité Européenne (CE) marked and one WHO prequalified rapid diagnostic tests in Uganda
Source: PLoS One. 2026 Apr 8;21(4):e0346259. doi: 10.1371/journal.pone.0346259 (PMC13061167; doi:10.1371/journal.pone.0346259)

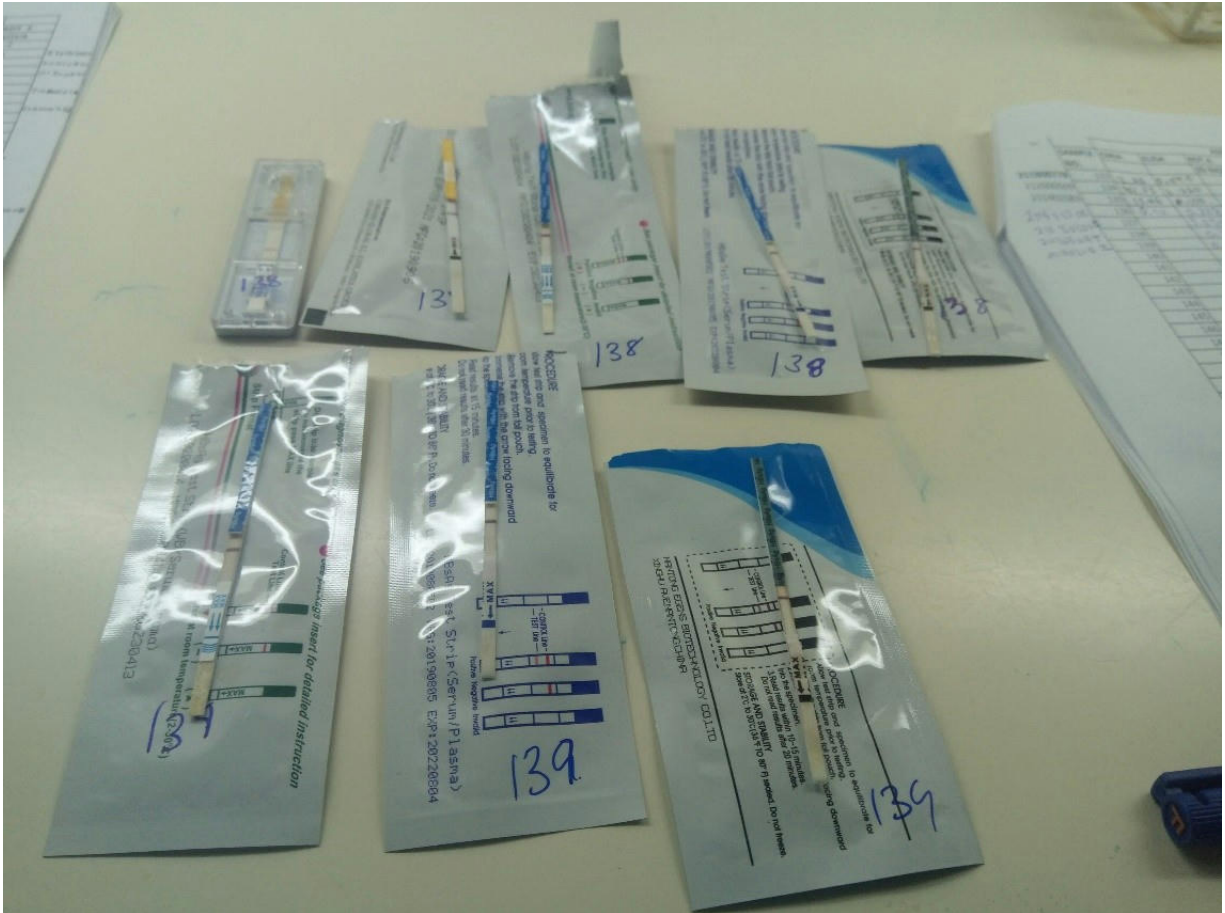

Figure1.tif

Supplement: S1 Fig — (PDF) [file pone.0346259.s001.pdf]
