# Supplementary material for: Diagnostic performance of four hepatitis-B surface antigen conformité Européenne (CE) marked and one WHO prequalified rapid diagnostic tests in Uganda
Source: PLoS One. 2026 Apr 8;21(4):e0346259. doi: 10.1371/journal.pone.0346259 (PMC13061167; doi:10.1371/journal.pone.0346259)
Supplement: S1 Appendix — (DOCX) [file pone.0346259.s002.docx]

**Appendix**

**ELISA laboratory procedure**

According to Fortress Diagnostic (2016, 30), this ELISA for HBsAg detection uses the antibody sandwich method. On the ELISA plate, three wells were marked for the negative control, and two wells for the positive control. One well was designated as the blank. Following the protocol, 20μL of diluent was added to each well except the blank. 100μL control and samples were then added. The plate was covered with the sealer provided in the kit and incubated for 60 minutes at 37◦C. The plate was removed, and 50μL HRP conjugate was added to each well except the blank and mixed by gently tapping the plate. The plate was then covered with a sealer and incubated for an additional 30 minutes, after which it was removed from the incubator and washed using phosphate buffer solution (PBS), for 5 cycles, each time while allowing the micro wells to soak for 30-60 seconds. After the washing cycle, the ELISA plate was turned down onto blotting paper and gently tapped to remove any remainders. 50 μL of chromogen A was added, followed by chromogen B to each well, including the blank. The plate was further incubated at 37◦C for 30 minutes. The enzymatic reaction between the chromogen solutions and the HRP- conjugate produced a blue color in the positive control and HBsAg-positive wells. 50 μL of stop solution was added to each well and mixed. Intensive yellow color developed in the positive control and HBsAg-positive sample wells. The optical density (OD value) of each well was determined at once with a microplate reader set to 630 nm. Generally, the results were interpreted as non-reactive when results were less than 1.00 and reactive when results were equal to or greater than 1.00; results were considered borderline between 0.9 and 1.1.
