# Supplementary material for: Diagnostic performance of four hepatitis-B surface antigen conformité Européenne (CE) marked and one WHO prequalified rapid diagnostic tests in Uganda
Source: PLoS One. 2026 Apr 8;21(4):e0346259. doi: 10.1371/journal.pone.0346259 (PMC13061167; doi:10.1371/journal.pone.0346259)
Supplement: Attachment — Submitted filename: Response to Reviewers.docx [file pone.0346259.s005.docx]

PONE-D-25-07381

Diagnostic Performance of Four Hepatitis-B Surface Antigen Conformité Européenne (CE) Marked and One WHO Prequalified Rapid Diagnostic Tests in Uganda

PLOS ONE

Dear Dr. Baliruno,

Thank you for submitting your manuscript to PLOS ONE. After careful consideration, we feel that it has merit but does not fully meet PLOS ONE’s publication criteria as it currently stands. Therefore, we invite you to submit a revised version of the manuscript that addresses the points raised during the review process.

Please submit your revised manuscript by Jun 09 2025 11:59PM. If you will need more time than this to complete your revisions, please reply to this message or contact the journal office at [plosone@plos.org](mailto:plosone@plos.org). When you're ready to submit your revision, log on to <https://www.editorialmanager.com/pone/> and select the 'Submissions Needing Revision' folder to locate your manuscript file.

Please include the following items when submitting your revised manuscript:

- A rebuttal letter that responds to each point raised by the academic editor and reviewer(s). You should upload this letter as a separate file labeled 'Response to Reviewers'.
- A marked-up copy of your manuscript that highlights changes made to the original version. You should upload this as a separate file labeled 'Revised Manuscript with Track Changes'.
- An unmarked version of your revised paper without tracked changes. You should upload this as a separate file labeled 'Manuscript'.

If you would like to make changes to your financial disclosure, please include your updated statement in your cover letter. Guidelines for resubmitting your figure files are available below the reviewer comments at the end of this letter.

If applicable, we recommend that you deposit your laboratory protocols in [protocols.io](http://protocols.io) to enhance the reproducibility of your results. Protocols.io assigns your protocol its own identifier (DOI) so that it can be cited independently in the future. For instructions see: [https://journals.plos.org/plosone/s/submission-guidelines#loc-laboratory-protocols](https://track.editorialmanager.com/CL0/https:%2F%2Fjournals.plos.org%2Fplosone%2Fs%2Fsubmission-guidelines%23loc-laboratory-protocols/1/010f01966f95d3da-911d5157-7535-408f-ae8d-dc5475403d15-000000/KkUPAu0THyQhnMfvbHC9bPb2lMNRSbOUDhFn9L2_c6Y=208). Additionally, PLOS ONE offers an option for publishing peer-reviewed Lab Protocol articles, which describe protocols hosted on [protocols.io](http://protocols.io). Read more information on sharing protocols at [https://plos.org/protocols?utm_medium=editorial-email&utm_source=authorletters&utm_campaign=protocols](https://track.editorialmanager.com/CL0/https:%2F%2Fplos.org%2Fprotocols%3Futm_medium=editorial-email%26utm_source=authorletters%26utm_campaign=protocols/1/010f01966f95d3da-911d5157-7535-408f-ae8d-dc5475403d15-000000/TXZBMKmbZnG7nEIde4LdDYSTnAGyACeQ-JGPe12mgwQ=208).

We look forward to receiving your revised manuscript.

Kind regards,

Seth Agyei Domfeh, PhD
Academic Editor
PLOS ONE

Journal requirements:

1. When submitting your revision, we need you to address these additional requirements.

Please ensure that your manuscript meets PLOS ONE's style requirements, including those for file naming. The PLOS ONE style templates can be found at

<https://journals.plos.org/plosone/s/file?id=wjVg/PLOSOne_formatting_sample_main_body.pdf> and

[https://journals.plos.org/plosone/s/file?id=ba62/PLOSOne_formatting_sample_title_authors_affiliations.pdf](https://track.editorialmanager.com/CL0/https:%2F%2Fjournals.plos.org%2Fplosone%2Fs%2Ffile%3Fid=ba62%2FPLOSOne_formatting_sample_title_authors_affiliations.pdf/1/010f01966f95d3da-911d5157-7535-408f-ae8d-dc5475403d15-000000/bqJ5CfNh9X9OzEZZqcEN8NuNbG_OzOau-kXepPxsRjY=208)

2. In the online submission form, you indicated that [The data underlying the results presented in the study are available from the corresponding author].

All PLOS journals now require all data underlying the findings described in their manuscript to be freely available to other researchers, either 1. In a public repository, 2. Within the manuscript itself, or 3. Uploaded as supplementary information.

This policy applies to all data except where public deposition would breach compliance with the protocol approved by your research ethics board. If your data cannot be made publicly available for ethical or legal reasons (e.g., public availability would compromise patient privacy), please explain your reasons on resubmission and your exemption request will be escalated for approval.

Reviewers' comments:

Reviewer's Responses to Questions

**Comments to the Author**

1. Is the manuscript technically sound, and do the data support the conclusions?

The manuscript must describe a technically sound piece of scientific research with data that supports the conclusions. Experiments must have been conducted rigorously, with appropriate controls, replication, and sample sizes. The conclusions must be drawn appropriately based on the data presented.

Reviewer #1: Yes

Reviewer #2: No

2. Has the statistical analysis been performed appropriately and rigorously?

Reviewer #1: Yes

Reviewer #2: No

3. Have the authors made all data underlying the findings in their manuscript fully available?

The [PLOS Data policy](http://track.editorialmanager.com/CL0/http:%2F%2Fwww.plosone.org%2Fstatic%2Fpolicies.action%23sharing/1/010f01966f95d3da-911d5157-7535-408f-ae8d-dc5475403d15-000000/iKNmDNNlBy92X2HaDGtrSGLy6CkNZdWxgvDLO1Yboig=208) requires authors to make all data underlying the findings described in their manuscript fully available without restriction, with rare exception (please refer to the Data Availability Statement in the manuscript PDF file). The data should be provided as part of the manuscript or its supporting information, or deposited to a public repository. For example, in addition to summary statistics, the data points behind means, medians and variance measures should be available. If there are restrictions on publicly sharing data—e.g. participant privacy or use of data from a third party—those must be specified.

Reviewer #1: Yes

Reviewer #2: Yes

4. Is the manuscript presented in an intelligible fashion and written in standard English?

PLOS ONE does not copyedit accepted manuscripts, so the language in submitted articles must be clear, correct, and unambiguous. Any typographical or grammatical errors should be corrected at revision, so please note any specific errors here.

Reviewer #1: Yes

Reviewer #2: Yes

5. Review Comments to the Author

Please use the space provided to explain your answers to the questions above. You may also include additional comments for the author, including concerns about dual publication, research ethics, or publication ethics. (Please upload your review as an attachment if it exceeds 20,000 characters)

Reviewer #1: Thank you for the effort on this well written paper addressing a very important concern on limited screening tests for hepatitis in a population which has substantive exposure. I have no concern with regards to dual publication, research ethics, or publication ethics.
Please see below for few comments which you can take into consideration as you work towards a final version:

**Comment:** In the abstract result section and the whole of the paper there is a result on sensitivity of 93% with a CI of 93 - 93%. Please confirm this reporting version.

**Response: Thank you for this comment. Yes, this is the reporting version.**

**Comment:** The cheaper RDTs here written as 'CE marked' compared with 'CE Marked' elsewhere in the document. Please consider for consistency

**Response: Thank you for this comment. This** **has been revised in the manuscript.**

**Introduction**

**Comment:** Line 40 please consider deleting the word 'above'

**Response: Thank you for this comment. This** **has been revised in the manuscript.**

**Comment:** Line 51 - 53 is this referring to only the Iranian study? Why is this part separated from the paragraph below which is addressing results from other studies?

**Response: Thank you for this comment. The Iranian study has been replaced with references to a meta-analysis, “Diagnostic accuracy of tests to detect hepatitis B surface antigen: a systematic review of the literature and meta-analysis”**

**Comment:** Line 60 please consider a new paragraph

**Response: Thank you for this comment. This has been revised in the manuscript.**

**Comment:** Line 68 – 70 please clarify if this is to detect acute infection

**Response: Thank you for the comment. The statement has been removed because RDTs can detect both acute and chronic hepatitis B surface antigen (HbsAg).**

**Methods and Materials**

**Comment:** Is 200 calculation for both positives and negatives? or for each?

**Response: Thank you for this comment. The 200 calculation is for both positive and negative samples.**

**Comment:** Is there any concern with multiple operators in the testing?

**Response: Thank you for this comment. Multiple operators were employed to reduce the risk of bias.**

**Results**

**Comment:** In the flow chart how many participants were not included due to heamolysis for example or the flow chart starts at the ones selected for the main batch of samples?
The flow chart itself may require redoing so that it is clearer and without any overlaps

**Response: Thank you for this comment. The flow chart has been removed, as it did not add any substantial value.**

**Comments:** In the tables it is noted that one decimal place and 2 decimals places are used

**Response: Thank you for this comment. This has been revised in the manuscript to one decimal place to ensure uniformity.**

**Appendix**

**Comment:** consider pictures of one each of the tests for clarity and submit as supplement. Add a description of the attachments

**Response: Thank you for the comment. However, not all the pictures for each one of the tests is available. For the available picture, I have added a description.**

Reviewer #2: This diagnostic performance evaluation tested four HBsAg CE marked and one WHO PQ RDTs in a blood transfusion service in Uganda.

The authors evaluated 200 residual blood donor samples, with a 1:1 ratio of known positive to negative samples.
Major comments:
**1. Comment:** Abstract. Please state the reference ELISA and RDT manufacturers. If additional space is required, the reference to the stats program could be removed.

**Response: Thank you for this comment. This has been revised in the abstract and the manuscript.**

**2. Comment:** Mention in the abstract the prevalence in population (e.g. 50% 1:1 case: control matching)

**Response: Thank you for this comment. This has been revised accordingly in the abstract.**

Introduction
**3. Comment:** The authors should provide a reference for this statement:
"Currently, the global mortality from viral 31 hepatitis exceeds that of HIV, TB or malaria, and is likely to exceed the toll from those three 32 diseases combined by 2040."

**Response: Thank you for this comment. The reference for the above statement has been added to the manuscript.**

**4. Comment:** The epidemiological estimates should be updated using the WHO global hepatitis report 2024.

**Response: Thank you for this comment. This is kindly updated and revised using the WHO global hepatitis report 2024.**

**5. Comment:** "Several immunological methods are available to detect HBsAg, including Enzyme Immuno Assays (EIA), Radio Immuno Assays (RIA), Immuno Chromatographic Assays (ICA), and Haemo Agglutination Assays (HAA)."
Both RIA and HAA are archaic methods and should not be refered to. Chemiluminescent immunoassays and EIAs are the leading methods. Immuno Chromatographic assays are commonly referred to as lateral flow or rapid diagnostic tests.

**Response: Thank you, this is kindly noted and revised in the manuscript.**
**6. Comment:** "In Iran, Acon, Atlas, Intec, Blue Cross, Dima and Cortez had sensitivities ranging from 97.5% -99.0 % and specificities of 97.5%- 99.2% (8). Varied performance characteristics were however, recorded in East and West African populations."

This study in Iran should not be referred to in the introduction and is not clearly relevant to the current study. There are more relevant data from Africa. The statement about data from East and West African populations is more relevant but belongs as an introduction to the following paragraph. I would recommend referring to a meta-analysis or review of HBsAg RDT performance.

**Response:** Thank you for this comment. The Iranian study has been replaced with references to a meta-analysis “Diagnostic accuracy of tests to detect hepatitis B surface antigen: a systematic review of the literature and meta-analysis”

**7. Comment:** When mentioning test kits, please refer to the manufacturer and city/country of origin, not just the brand name (there are many similar brands of kits).

**Response: Thank you for the comment, this is kindly noted and revised.**

**8. Comment:** "The WHO
62 prequalified diagnostic kits are recommended internationally, however, standards for rapid
63 diagnostic test prequalification by this organization are quite stringent, making them expensive.
64 In Sub-Saharan Africa, developing countries such as Uganda, use standards for validating
65 diagnostic tools set by international regulatory bodies. These bodies include: the United States
66 Food and Drug Administration and the Conformité Européenne (CE) that are less costly as a
67 precondition for considering a diagnostic test"

I am not aware of any evidence that FDA or CE evaluation is less rigorous than the WHO PQ (or less costly). The reference provided is "ASLM. Lab culture, The ASLM Magazine. 2015;", which does not refer to a specific article (no URL or page number) or provide evidence for this assertion.

**Response: Thank you for your comment. I have updated the reference (12) alluding to the statement above, because the earlier reference was removed from the website.**

8. Comment: "However, for acute hepatitis B, RDTs may not be suitable since they can provide a false sense of security in case of negativity (14)."
This is not the case, HBsAg is positive during acute hepatitis, the reference 14 refers to national testing guidelines and not any empirical evidence to back this assertion.

**Response: Thank you for the comment. The statement has been removed from the manuscript following the guidance provided.**

**9. Comment:** "We therefore, aimed to calculate the sensitivity, specificity, positive and negative predictive values of 4 brands of RDTs in Uganda."
I believe you tested 5 brands?

**Response: Thank you for this comment. Yes, we tested 5 brands, and this has been corrected in the manuscript.**

**10. Comment:** The sample size formula refers to a national prevalence of 4.1% but this has not been used to calculate the sample size, instead a prevalence of 50% has been used in keeping with the study design.

**Response: Thank you for this comment. This has been kindly noted.**

**11. Comment:** For the Fortress diagnostic ELISA reference test, please state any available data on diagnostic/analytical sensitivity, validation data, and stringent regulatory approvals for this assay.

**Response:** Thank you for this comment. I have added to the available details for the Fortress diagnostic ELISA reference test in the manuscript.

**12. Comment:** The details on methods for ELISA are not necessary, they can be moved to an appendix and just state according to manufacturer’s instructions.

**Response: Thank you for comment. The details on methods for ELISA have been moved to the appendix.**

**13. Comment:** Was the result of the ELISA test available to the operator doing the RDT assessments? If so, state this and list as a limitation (unblinded and not independent assessment weakens the study quality).

**Response: The ELISA result was available to the operator doing the RDT and I have stated this as a limitation in the limitations section.**

**14. Comments:** The method to retest multiple times in case of discordance with the ELISA result has resulted in an artificially (improved) assessment of test performance in relation to real conditions. This is a concern, this renders the resulting performance characteristics essentially invalid.

"Retesting was done where results were discordant, this was done to improve the accuracy of the study results"
This is inappropriate. If available, present the results before retesting multiple times ie. the first test result.

**Response: Thank you for the comment. The wording may have implied the manipulation of the result. However, this was not the case an internal rapid diagnostic validation protocol was followed.**

15. Comments: The flow chart is not interpretable, the lines are crossed and do not flow properly. Since the flowchart does not show any study exclusions, it is better summarised in a table (as shown in table 3).

**Response: Thank you for the comment. The flow chart was removed as it did not add any substantial value to the study.**

**16. Comments:** Stratify table 1 by HBsAg test result, as well as showing overall characteristics.

**Response: Thank you for the comment. Overall characteristics are shown in the table. Initially the table was stratified by gender, but this was revised. However, I forgot to correct the heading, which has been corrected.**

17. A major consideration is that by taking a 1:1 case: control ratio, the sample prevalence is 50% vs 4% nationally. Therefore all predictive values presented (PPV and NPV) are invalid, they are wildly different from a scenario when the prevalence is 4%. I would not present any predictive values. If these are to be shown, it should be shown as simulation data, weighting for a hypothetical scenario with sample prevalence of 4% (and where test performance in positive and negative samples is applied to the hypothetical scenario).

**Response: Thank you for the comment. The positive and negative predictive values that are presented have been removed from the manuscript.**

6. Comment: PLOS authors have the option to publish the peer review history of their article ([what does this mean?](https://track.editorialmanager.com/CL0/https:%2F%2Fjournals.plos.org%2Fplosone%2Fs%2Feditorial-and-peer-review-process%23loc-peer-review-history/1/010f01966f95d3da-911d5157-7535-408f-ae8d-dc5475403d15-000000/TVRwcB5QxTI9h1jfqaKJYYtGxyHF8mUtlyV1haKxSgM=208)). If published, this will include your full peer review and any attached files.


If you choose “no”, your identity will remain anonymous but your review may still be made public.

**Response: No**


**Do you want your identity to be public for this peer review?** For information about this choice, including consent withdrawal, please see our [Privacy Policy](https://track.editorialmanager.com/CL0/https:%2F%2Fwww.plos.org%2Fprivacy-policy/1/010f01966f95d3da-911d5157-7535-408f-ae8d-dc5475403d15-000000/ND51--2q2Ob0-7jl8ylsM_Lw33CP7uuJMJwIND_DnuM=208).

Reviewer #1: No

Reviewer #2: No

I look forward to your continued support and please do not hesitate to contact me for further clarification

Sincerely,

Leah Naluwagga Baliruno
