# Supplementary material for: Diagnostic performance of four hepatitis-B surface antigen conformité Européenne (CE) marked and one WHO prequalified rapid diagnostic tests in Uganda
Source: PLoS One. 2026 Apr 8;21(4):e0346259. doi: 10.1371/journal.pone.0346259 (PMC13061167; doi:10.1371/journal.pone.0346259)
Supplement: Attachment — Submitted filename: Response to reviewers_PLOS One manuscript_10-12-25_LN.docx [file pone.0346259.s006.docx]

PONE-D-25-07381R1

Diagnostic Performance of Four Hepatitis-B Surface Antigen Conformité Européenne (CE) Marked and One WHO Prequalified Rapid Diagnostic Tests in Uganda

PLOS ONE

Dear Dr. Baliruno,

Thank you for submitting your manuscript to PLOS ONE. After careful consideration, we feel that it has merit but does not fully meet PLOS ONE’s publication criteria as it currently stands. Therefore, we invite you to submit a revised version of the manuscript that addresses the points raised during the review process.

Please submit your revised manuscript by Nov 23 2025 11:59PM. If you will need more time than this to complete your revisions, please reply to this message or contact the journal office at [plosone@plos.org](mailto:plosone@plos.org). When you're ready to submit your revision, log on to <https://www.editorialmanager.com/pone/> and select the 'Submissions Needing Revision' folder to locate your manuscript file.

Please include the following items when submitting your revised manuscript:

- A rebuttal letter that responds to each point raised by the academic editor and reviewer(s). You should upload this letter as a separate file labeled 'Response to Reviewers'.
- A marked-up copy of your manuscript that highlights changes made to the original version. You should upload this as a separate file labeled 'Revised Manuscript with Track Changes'.
- An unmarked version of your revised paper without tracked changes. You should upload this as a separate file labeled 'Manuscript'.

If you would like to make changes to your financial disclosure, please include your updated statement in your cover letter. Guidelines for resubmitting your figure files are available below the reviewer comments at the end of this letter.

If applicable, we recommend that you deposit your laboratory protocols in [protocols.io](http://protocols.io) to enhance the reproducibility of your results. Protocols.io assigns your protocol its own identifier (DOI) so that it can be cited independently in the future. For instructions see: [https://journals.plos.org/plosone/s/submission-guidelines#loc-laboratory-protocols](https://track.editorialmanager.com/CL0/https:%2F%2Fjournals.plos.org%2Fplosone%2Fs%2Fsubmission-guidelines%23loc-laboratory-protocols/1/010f0199cbbf8beb-a50b09e7-cc64-4b8b-a977-b78ec6f307ea-000000/1z0_UmQiU3Ox-5yljMwoJxLVIXGMMo4SEnp7qawBARc=232). Additionally, PLOS ONE offers an option for publishing peer-reviewed Lab Protocol articles, which describe protocols hosted on [protocols.io](http://protocols.io). Read more information on sharing protocols at [https://plos.org/protocols?utm_medium=editorial-email&utm_source=authorletters&utm_campaign=protocols](https://track.editorialmanager.com/CL0/https:%2F%2Fplos.org%2Fprotocols%3Futm_medium=editorial-email%26utm_source=authorletters%26utm_campaign=protocols/1/010f0199cbbf8beb-a50b09e7-cc64-4b8b-a977-b78ec6f307ea-000000/Ilbv7WrJeJ1Y7GzyFCnfrBCXCxfT69_CypRYQjHBbMQ=232).

We look forward to receiving your revised manuscript.

Kind regards,

Seth Agyei Domfeh, PhD
Academic Editor
PLOS ONE

Journal Requirements:

If the reviewer comments include a recommendation to cite specific previously published works, please review and evaluate these publications to determine whether they are relevant and should be cited. There is no requirement to cite these works unless the editor has indicated otherwise. 


[Note: HTML markup is below. Please do not edit.]

Reviewers' comments:

Reviewer's Responses to Questions

**Comments to the Author**

1. If the authors have adequately addressed your comments raised in a previous round of review and you feel that this manuscript is now acceptable for publication, you may indicate that here to bypass the “Comments to the Author” section, enter your conflict of interest statement in the “Confidential to Editor” section, and submit your "Accept" recommendation.

Reviewer #2: (No Response)

2. Is the manuscript technically sound, and do the data support the conclusions?

The manuscript must describe a technically sound piece of scientific research with data that supports the conclusions. Experiments must have been conducted rigorously, with appropriate controls, replication, and sample sizes. The conclusions must be drawn appropriately based on the data presented.

Reviewer #2: No

3. Has the statistical analysis been performed appropriately and rigorously?

Reviewer #2: No

4. Have the authors made all data underlying the findings in their manuscript fully available?

The [PLOS Data policy](http://track.editorialmanager.com/CL0/http:%2F%2Fwww.plosone.org%2Fstatic%2Fpolicies.action%23sharing/1/010f0199cbbf8beb-a50b09e7-cc64-4b8b-a977-b78ec6f307ea-000000/jPhHR985Z00b4z6QMqM780cgBtTlWJRmnKzvDryS-UY=232) requires authors to make all data underlying the findings described in their manuscript fully available without restriction, with rare exception (please refer to the Data Availability Statement in the manuscript PDF file). The data should be provided as part of the manuscript or its supporting information, or deposited to a public repository. For example, in addition to summary statistics, the data points behind means, medians and variance measures should be available. If there are restrictions on publicly sharing data—e.g. participant privacy or use of data from a third party—those must be specified.

Reviewer #2: Yes

5. Is the manuscript presented in an intelligible fashion and written in standard English?

PLOS ONE does not copyedit accepted manuscripts, so the language in submitted articles must be clear, correct, and unambiguous. Any typographical or grammatical errors should be corrected at revision, so please note any specific errors here.

Reviewer #2: Yes

6. Review Comments to the Author

Please use the space provided to explain your answers to the questions above. You may also include additional comments for the author, including concerns about dual publication, research ethics, or publication ethics. (Please upload your review as an attachment if it exceeds 20,000 characters)

Reviewer #2: There are some unresolved issues that require revision:

1. The abstract continues to refer to 296 million people with HBV, while the introduction has been updated with Global Hepatitis report data from WHO (254 million). Please be consistent.

**Thank you for the comment. I have adjusted this accordingly.**

2. The abstract refers to SD bioline as having superior specificity relative to the other assays but the confidence intervals are entirely overlapping. I would recommend recharacterizing this as all assays having good specificity, as the finding for SD bioline may be a chance finding.

**Thank you, this is well noted. I have rectified this.**

3. Introduction:
"An estimated 254 million people are living with hepatitis B. Only 13%
35 of people living with chronic hepatitis B infection had been diagnosed, presenting a big gap in
36 testing for HBV, and close to 3% had received antiviral therapy at the end of 2022. The WHO
37 African Region accounts for 63% of new hepatitis B infections (3)."

I would recommend to report the statistics for Africa from this report (4% diagnosed and 0.2% treated).

**Thank you for this comment. The statistics have been adjusted to reflect the current HBV status.**

4. "Several immunological methods are available."
This isn't strictly an immunological method since we are detecting viral antigens, not antibodies. I would recharacterize as "serological methods" instead.
Here I would also mention chemiluminescent immunoassays (CLIA) as this is the most commonly used method globally.

5. "The WHO prequalified diagnostic kits are recommended
63 internationally, however, standards for rapid diagnostic test prequalification by this organization
64 are quite stringent, making them expensive."

Again, I am making the same point here: I do not think that the WHO PQ is more stringent than CE marking or FDA approval. I believe it is the opposite, fees for FDA or CE marking can exceed 200k USD whereas fees for WHO PQ are substantially lower and may be abridged if the product already has stringent regulatory approval. The WHO PQ process may be slower and requires an independent performance evaluation but to my knowledge there is no evidence it is more stringent. The cited article does not provide any evidence in support of the assertion made here.

**Thank you for this comment. This statement has been removed.**

6. Again I am making the same point here: The sample size calculation again makes reference to the Uganda local prevalence, which is not relevant, since a 50% prevalence was used in the study design. The authors used a study which showed a prevalence of 45% as the source estimate- surely this cannot be considered as the expected sensitivity. I would recalculate the sample size using a more realistic sensitivity estimate (eg. from the cited meta-analyiss) and include the prevalence of 50% as per the study design, as the current sample size calculation is not credible.

**Thank you for your comment. I have used the average sensitivity 98.9% and average specificity of 96.7% from a recent meta-analysis conducted and the local prevalence of hepatitis among local blood donors in Uganda. Using 50% the sample size was too low for diagnostic accuracy.**

7. Again I am making the same point here: again, the study design refers to retesting if there was discrepancy, and then taking only the final result from 3 tests to evaluate at the final result in the event of discrepancy.

At the risk of repeating myself, this is not an appropriate study design since it artificially inflates the diagnostic performance. Instead, take the result from the first RDT result only (disregard the outcome of repeated testing) and use this to evaluate performance. You may later specify the result of retesting in a separate section, but do not include this in the diagnostic test evaluation.

**Thank you for this comment. I have considered the first RDT test result and have removed the statement from the diagnostic test evaluation.**

8. Is there any room to state what is the HBsAg prevalence in blood donors locally?

**Thank you for the comment. I have added the HBsAg prevalence of blood donors locally, which ranges from 2.8% to 4.1% among blood donors in Uganda, under “sample size determination and sample collection”.**

9. For the 7 samples which were negative with all 5 RDTs, was there a systematic difference observed in S/CO in the ELISA result eg, close to 1? Were ELISA results checked in duplicate?

**Thank you for the comment. The ELISA results were checked in duplicate, and the S/CO was consistently below 1.0.**

10. Discussion:
"SD Bioline, the WHO prequalified kit, had the best diagnostic performance." Again see point above about the lack of statistical difference in specificity for this test vs the others.

**Thank you for your comment. I have removed this statement from the discussion.**

11. "False-negative results are a potential vehicle of silent transmit
186 ssion and spread of disease infection among people (21)."
Check spelling of "transmission" and grammar of "disease infection"

**Thank you for this comment. This has been noted and corrected accordingly.**

12. Add to the limitations that the study design used 1:1 matching meaning that the predictive values were not interpretable for this population.

**Thank you for this comment. I have added this to the limitations.**

13. I would add to the interpretation that the RDTs were found not to be suitable for a blood donor screening population, as the sensitivity was insufficient.

**Thank you for this comment. I have also as part of the limitations.**

7. PLOS authors have the option to publish the peer review history of their article ([what does this mean?](https://track.editorialmanager.com/CL0/https:%2F%2Fjournals.plos.org%2Fplosone%2Fs%2Feditorial-and-peer-review-process%23loc-peer-review-history/1/010f0199cbbf8beb-a50b09e7-cc64-4b8b-a977-b78ec6f307ea-000000/1Vo1midPZvr8j_UliqVHO7p1oscSpXwHDMRJVmZ56aw=232)). If published, this will include your full peer review and any attached files.


If you choose “no”, your identity will remain anonymous but your review may still be made public.


**Do you want your identity to be public for this peer review?** For information about this choice, including consent withdrawal, please see our [Privacy Policy](https://track.editorialmanager.com/CL0/https:%2F%2Fwww.plos.org%2Fprivacy-policy/1/010f0199cbbf8beb-a50b09e7-cc64-4b8b-a977-b78ec6f307ea-000000/rOYiD1vHMqAb5b03K574hXasfc-xRLFFjdpxyTRvtCQ=232).

Reviewer #2: No

[NOTE: If reviewer comments were submitted as an attachment file, they will be attached to this email and accessible via the submission site. Please log into your account, locate the manuscript record, and check for the action link "View Attachments". If this link does not appear, there are no attachment files.]

While revising your submission, please upload your figure files to the Preflight Analysis and Conversion Engine (PACE) digital diagnostic tool, [https://pacev2.apexcovantage.com/](https://track.editorialmanager.com/CL0/https:%2F%2Fpacev2.apexcovantage.com%2F/1/010f0199cbbf8beb-a50b09e7-cc64-4b8b-a977-b78ec6f307ea-000000/gI2ebf9FBlP3mP3YQy29-LgdMs72SUyh1DiInzREqsc=232). PACE helps ensure that figures meet PLOS requirements. To use PACE, you must first register as a user. Registration is free. Then, login and navigate to the UPLOAD tab, where you will find detailed instructions on how to use the tool. If you encounter any issues or have any questions when using PACE, please email PLOS at [figures@plos.org](mailto:figures@plos.org). Please note that Supporting Information files do not need this step.
