# Supplementary material for: Diagnostic performance of four hepatitis-B surface antigen conformité Européenne (CE) marked and one WHO prequalified rapid diagnostic tests in Uganda
Source: PLoS One. 2026 Apr 8;21(4):e0346259. doi: 10.1371/journal.pone.0346259 (PMC13061167; doi:10.1371/journal.pone.0346259)
Supplement: Attachment — Submitted filename: Response to Reviewers_11-03-2026_LN.docx [file pone.0346259.s007.docx]

PONE-D-25-07381R2

Diagnostic Performance of Four Hepatitis-B Surface Antigen Conformité Européenne (CE) Marked and One WHO Prequalified Rapid Diagnostic Tests in Uganda

PLOS One

Dear Dr. Baliruno,

Thank you for submitting your manuscript to PLOS ONE. After careful consideration, we feel that it has merit but does not fully meet PLOS ONE’s publication criteria as it currently stands. Therefore, we invite you to submit a revised version of the manuscript that addresses the points raised during the review process.

Please submit your revised manuscript by Mar 27 2026 11:59PM. If you will need more time than this to complete your revisions, please reply to this message or contact the journal office at [plosone@plos.org](mailto:plosone@plos.org). When you're ready to submit your revision, log on to <https://www.editorialmanager.com/pone/> and select the 'Submissions Needing Revision' folder to locate your manuscript file.

Please include the following items when submitting your revised manuscript:

- A letter that responds to each point raised by the academic editor and reviewer(s). You should upload this letter as a separate file labeled 'Response to Reviewers'.
- A marked-up copy of your manuscript that highlights changes made to the original version. You should upload this as a separate file labeled 'Revised Manuscript with Track Changes'.
- An unmarked version of your revised paper without tracked changes. You should upload this as a separate file labeled 'Manuscript'.

If you would like to make changes to your financial disclosure, please include your updated statement in your cover letter. Guidelines for resubmitting your figure files are available below the reviewer comments at the end of this letter.

If applicable, we recommend that you deposit your laboratory protocols in [protocols.io](http://protocols.io) to enhance the reproducibility of your results. Protocols.io assigns your protocol its own identifier (DOI) so that it can be cited independently in the future. For instructions see: [https://journals.plos.org/plosone/s/submission-guidelines#loc-laboratory-protocols](https://track.editorialmanager.com/CL0/https:%2F%2Fjournals.plos.org%2Fplosone%2Fs%2Fsubmission-guidelines%23loc-laboratory-protocols/1/010f019c474db65b-71dabe52-6a04-46bc-9591-76219b09642d-000000/1eT-lTvWSQ6-R5Zk6h2Wjj3nMS03K4SzxPgDB-d02P4=249). Additionally, PLOS ONE offers an option for publishing peer-reviewed Lab Protocol articles, which describe protocols hosted on [protocols.io](http://protocols.io). Read more information on sharing protocols at [https://plos.org/protocols?utm_medium=editorial-email&utm_source=authorletters&utm_campaign=protocols](https://track.editorialmanager.com/CL0/https:%2F%2Fplos.org%2Fprotocols%3Futm_medium=editorial-email%26utm_source=authorletters%26utm_campaign=protocols/1/010f019c474db65b-71dabe52-6a04-46bc-9591-76219b09642d-000000/bontp7_SJec5_s5o9R_3Fikit7jVSDmWl6dNTZhZNGw=249).

We look forward to receiving your revised manuscript.

Kind regards,

Seth Agyei Domfeh, PhD
Academic Editor
PLOS One

Journal Requirements:

If the reviewer comments include a recommendation to cite specific previously published works, please review and evaluate these publications to determine whether they are relevant and should be cited. There is no requirement to cite these works unless the editor has indicated otherwise. 

Please review your reference list to ensure that it is complete and correct. If you have cited papers that have been retracted, please include the rationale for doing so in the manuscript text, or remove these references and replace them with relevant current references. Any changes to the reference list should be mentioned in the rebuttal letter that accompanies your revised manuscript. If you need to cite a retracted article, indicate the article’s retracted status in the References list and also include a citation and full reference for the retraction notice.


[Note: HTML markup is below. Please do not edit.]

Reviewers' comments:

Reviewer's Responses to Questions

**Comments to the Author**

1. If the authors have adequately addressed your comments raised in a previous round of review and you feel that this manuscript is now acceptable for publication, you may indicate that here to bypass the “Comments to the Author” section, enter your conflict of interest statement in the “Confidential to Editor” section, and submit your "Accept" recommendation.

Reviewer #2: (No Response)

2. Is the manuscript technically sound, and do the data support the conclusions?

The manuscript must describe a technically sound piece of scientific research with data that supports the conclusions. Experiments must have been conducted rigorously, with appropriate controls, replication, and sample sizes. The conclusions must be drawn appropriately based on the data presented.

Reviewer #2: Partly

3. Has the statistical analysis been performed appropriately and rigorously?

Reviewer #2: Yes

4. Have the authors made all data underlying the findings in their manuscript fully available?

The [PLOS Data policy](http://track.editorialmanager.com/CL0/http:%2F%2Fwww.plosone.org%2Fstatic%2Fpolicies.action%23sharing/1/010f019c474db65b-71dabe52-6a04-46bc-9591-76219b09642d-000000/yezAXZ7e_Q98bjAkPpJPqlO07F1PEnbUOU1CtQq9y_c=249) requires authors to make all data underlying the findings described in their manuscript fully available without restriction, with rare exception (please refer to the Data Availability Statement in the manuscript PDF file). The data should be provided as part of the manuscript or its supporting information, or deposited to a public repository. For example, in addition to summary statistics, the data points behind means, medians and variance measures should be available. If there are restrictions on publicly sharing data—e.g. participant privacy or use of data from a third party—those must be specified.

Reviewer #2: Yes

5. Is the manuscript presented in an intelligible fashion and written in standard English?

PLOS ONE does not copyedit accepted manuscripts, so the language in submitted articles must be clear, correct, and unambiguous. Any typographical or grammatical errors should be corrected at revision, so please note any specific errors here.

Reviewer #2: Yes

6. Review Comments to the Author

Please use the space provided to explain your answers to the questions above. You may also include additional comments for the author, including concerns about dual publication, research ethics, or publication ethics. (Please upload your review as an attachment if it exceeds 20,000 characters)

Reviewer #2: The authors have addressed most of my comments from the previous revision. However, there are two outstanding issues:

1. In section 2.2 it continues to reference a prevalence of between 2.8 to 4.1% for calculating the sample size.
This is the third time I am making the same point. The study is a case:control design, the design prevalence is 50%, you cannot use the expected population prevalence (3-4%) to estimate your sample size, when the study prevalence is 50%.

The rationale given in the comments is effectively that the result was unwanted, which is unacceptable:
"Thank you for your comment. I have used the average sensitivity 98.9% and average specificity of 96.7% from a recent meta-analysis conducted and the local prevalence of hepatitis among local blood donors in Uganda. Using 50% the sample size was too low for diagnostic accuracy."

If I apply a sample size calculation in R:
library(SampleSizeDiagnostics)
\# Example assumptions
Se0 <- 0.95 # Round to more conservatives estimates
Sp0 <- 0.95
p <- 0.50 # Case control prevalence= 50%
CI <- 0.95
w <- 0.05 # Half-width of 5%
SampleSizeDiagnostics(sn = Se0, sp = Sp0, p = p, w=w, CI=CI)
Result: Sample size = 146

**Response**

**Thank you for the comment. I have taken into consideration the fact that this is a case-control study, and I have revised section 2.2 Sample size determination and sample collection accordingly.**

2. From the previous review:
"13. I would add to the interpretation that the RDTs were found not to be suitable for a blood
donor screening population, as the sensitivity was insufficient."

"Thank you for this comment. I have also as part of the limitations."

I cannot see this point added in the limitations. You mention the "window period" but the discussion does not specifically mention this important point that the observed sensitivity would be insufficient for blood donor screening according to WHO criteria, and low level HBsAg may been seen most commonly in people approaching spontaneous seroclearance/ functional cure.

**Response**

**Thank you for this comment. I have added this limitation in the write-up as advised.**

7. PLOS authors have the option to publish the peer review history of their article ([what does this mean?](https://track.editorialmanager.com/CL0/https:%2F%2Fjournals.plos.org%2Fplosone%2Fs%2Feditorial-and-peer-review-process%23loc-peer-review-history/1/010f019c474db65b-71dabe52-6a04-46bc-9591-76219b09642d-000000/jGhKv5QUzXi0jMUml2elRrLLI-w3FkrZwkjH0-lmtkU=249)). If published, this will include your full peer review and any attached files.

If you choose “no”, your identity will remain anonymous, but your review may still be made public.

**Do you want your identity to be public for this peer review?** For information about this choice, including consent withdrawal, please see our [Privacy Policy](https://track.editorialmanager.com/CL0/https:%2F%2Fwww.plos.org%2Fprivacy-policy/1/010f019c474db65b-71dabe52-6a04-46bc-9591-76219b09642d-000000/iTah_HkwGBwJDFPJW5faFSoa7OGY19ujhmdat63mH5g=249).

Reviewer #2: No

[NOTE: If reviewer comments were submitted as an attachment file, they will be attached to this email and accessible via the submission site. Please log into your account, locate the manuscript record, and check for the action link "View Attachments". If this link does not appear, there are no attachment files.]
